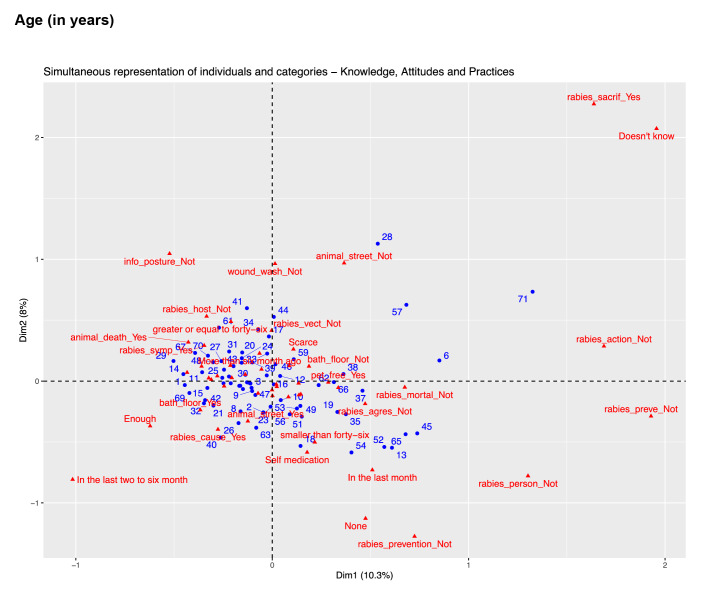# Knowledge, attitudes, and practices towards rabies: A preliminary cross-sectional appraisal in Colombia

**DOI:** 10.7705/biomedica.7161

**Published:** 2024-11-06

**Authors:** Samir Meriño-Olivella, María del Pilar Sánchez-Bonilla, Daniel Camilo Aguirre-Acevedo, Nathalia M. Correa-Valencia

**Affiliations:** 1 Grupo de Investigación Centauro, Escuela de Medicina Veterinaria, Facultad de Ciencias Agrarias, Universidad de Antioquia, Medellín, Colombia Universidad de Antioquia Grupo de Investigación Centauro, Escuela de Medicina Veterinaria Facultad de Ciencias Agrarias Universidad de Antioquia Medellín Colombia; 2 Grupo de Investigadores en Medicina y Producción Tropical Animal - IMPRONTA, Facultad de Medicina Veterinaria y Zootecnia, Universidad Cooperativa de Colombia, Ibagué, Colombia Universidad Cooperativa de Colombia Grupo de Investigadores en Medicina y Producción Tropical Animal - IMPRONTA Facultad de Medicina Veterinaria y Zootecnia Universidad Cooperativa de Colombia Ibagué Colombia; 3 Grupo Académico de Epidemiología Clínica, Instituto de Investigaciones Médicas, Facultad de Medicina, Universidad de Antioquia, Medellín, Colombia Universidad de Antioquia Instituto de Investigaciones Médicas Facultad de Medicina Universidad de Antioquia Medellín Colombia

**Keywords:** Health knowledge, attitudes, practice, rabies virus, Colombia, conocimientos, actitudes y prácticas en salud, virus de la rabia, Colombia

## Abstract

**Introducción.:**

El virus de la rabia causa una infección cerebral fatal en los mamíferos y cualquier especie es susceptible de sufrirla. Todo esfuerzo encaminado a reconocer animales infectados y ejecutar las primeras acciones en caso de transmisión es obligatorio.

**Objetivo.:**

Determinar los perfiles de conocimientos, actitudes y prácticas relacionadas con rabia, o hidrofobia, en un municipio de Colombia mediante análisis de correspondencia múltiple.

**Materiales y métodos.:**

Se realizó un estudio descriptivo observacional con 71 dueños de mascotas en el municipio de Ibagué, Tolima, Colombia. Mediante una encuesta, se recolectaron datos sobre conocimientos, actitudes y prácticas relacionadas con la rabia, además de datos demográficos. La encuesta se llevó a cabo entre octubre y noviembre del 2021, y el análisis de datos incluyó estadísticas descriptivas y el de correspondencia múltiple.

**Resultados.:**

El estudio reveló un nivel notable de conciencia sobre la infección por virus de la rabia entre los residentes urbanos del municipio de estudio. Sin embargo, se identificaron causas de preocupación, como que permitían el ingreso de animales callejeros a los hogares y la falta de conciencia sobre la importancia de notificar sobre animales fallecidos. Los encuestados demostraron un enfoque humano en el manejo de las mordeduras y enfatizaron la importancia de limpiar las heridas; también, expresaron un fuerte deseo de obtener más información para mejorar sus conocimientos sobre la enfermedad.

**Conclusión.:**

Los hallazgos de este estudio brindan conocimientos valiosos para mejorar los esfuerzos de prevención de la rabia o hidrofobia, y promover la salud pública. La educación en salud, las estrategias basadas en evidencia y la participación comunitaria, son fundamentales para el control exitoso de la enfermedad, así como para abordar las brechas educativas que incluyen factores socioculturales.

## Resumen

**Introduction.:**

Rabies virus infection can cause fatal brain disease in mammals. Any species is susceptible to infection. Any effort aimed at recognizing infected animals and performing first actions in the event of transmission is mandatory.

**Objective.:**

To determine knowledge, attitudes, and practice profiles regarding rabies, or hydrophobia, in a municipality of Colombia, using a multiple correspondence analysis.

**Materials and methods.:**

A descriptive observational study involving 71 pet owners was carried out in the municipality of Ibagué (Tolima). A questionnaire-based survey collected data on rabies knowledge, attitudes, practices, and demographic information. The survey was conducted between October and November 2021, and the data analysis involved descriptive statistics and multiple correspondence analysis.

**Results.:**

The study revealed a commendable level of rabies awareness among the urban residents in the study municipality. However, there are causes of concern as they allow stray animals to enter their homes and are not aware of the importance of notifying dead animals. The respondents demonstrated a humane approach to bite management and emphasized the importance of wound cleaning. Additionally, they expressed a strong desire for more information to enhance their knowledge and awareness of the disease.

**Conclusion.:**

The findings of this study provide valuable insights for improving rabies prevention efforts and promoting public health. Health education, evidence-based strategies, and community participation are essential for successful disease control and educational gaps addressing related to sociocultural factors.

Rabies virus (RABV) can cause a fatal brain infection in mammals, and any species is susceptible to infection ^1^. Rabies is considered a neglected tropical disease that mainly affects vulnerable or poor populations living in hard-to-reach rural areas ^2^.

Rabies is one of the diseases with the highest surveillance activities in the Americas. Every two years, as part of the REDIPRA framework (https://www.paho.org/es/panaftosa/redipra), each country -including Colombia-, works on the Regional Plan for the Prevention of Human Rabies to continue the process of eliminating dog-transmitted rabies and reducing the risk of rabies transmitted by wildlife species in the continent.

Although this zoonosis is a vaccine-preventable disease, it continues to have a significant impact on public health worldwide, causing more than 59,000 human deaths in developing countries, mainly in Asia and Africa. Eighty percent of the cases occur in rural areas, given the limited first-line medical care, immunoglobulins, and vaccines ^3^.

The natural viral infection is mostly transmitted by bats, responsible for most rabies cases in cats, dogs, and other animals ^4^. In humans, the virus can be spread through different types of exposure, including scratches, abrasions, or wounds exposed to saliva or other infectious material from an animal with the disease. From any point of view, any contact with a potentially rabies-transmitting animal should be avoided ^5^.

Since the 20^th^ century, different efforts have been made to eliminate rabies, primarily focused on dog-mediated transmission. The "Zero by 30", led by the World Health Organization (WHO) and proposed in 2018, is the most global strategy ^6^. In this sense, health education is vital for the community's empowerment when formulating and evaluating educational programs to prevent the disease and prioritize the guidelines not yet considered in populations at risk ^7^.

Knowledge, attitudes, and practices surveys are one of the most accepted tools worldwide for medical/public health-related study programs defining what is known, believed, and done about a particular topic and improving awareness and knowledge of diseases affecting public health. These surveys also help to identify cultural beliefs and behavioral gaps that can pose serious dangers and barriers, especially against zoonotic diseases. These strategies can be useful in public health awareness programs and disease control estimation, under the principle that greater knowledge will result in modified attitudes and practices, minimizing disease burden and contributing to environmental changing conditions against the disease ^8^.

Knowledge, attitudes, and practices (KAP)-based strategies can be used to organize different types of activities, such as public health awareness campaigns, and to generate a supply of reference data for the planning, application, and evaluation of national programs for disease control, including rabies ^9^.

The multiple correspondence analysis technique is a descriptive method to evaluate multiple categorical variables ^10^^,^^11^. The generation of indices or scales from self-reported information has been an increasing need in public health, and new methods and theories have become progressively widespread ^12^.

In this context, knowledge, attitudes, and practices surveys can provide an important amount of categorical data, and multiple correspondence analysis allows the connection of independent data sets to identify comparable trends among them ^13^. Therefore, this study aimed to determine knowledge, attitudes, and practice profiles regarding rabies in a municipality of Colombia using a multiple correspondence analysis.

## Materials and methods

### Study area and sample frame

We designed a questionnaire-based descriptive observational, cross-sectional study with an intentional, non-probabilistic, voluntary sampling. A population of 71 pet owners (cats and dogs) domiciled in Ibagué hills (La Martinica, Noroccidentales, Pan de Azúcar, Cerro Gordo) in the department of Tolima, Colombia, were targeted for the study. Such areas are environmentally preserved within the city, representing a distinctive blend of urban and rural landscapes. Here, domestic animals have direct or indirect contact with the wild animals inhabiting these territories. The selection of the study area and the number of participants were driven by logistical considerations, budget restrictions, and researchers' familiarity with the geographic area.

As inclusion criteria, participants must be of legal age according to Colombian law (> 18 years old), own at least one dog and one cat -as information on both species is required-, express their intention and willingness to participate in the study, and have completed and signed the informed consent. The survey was conducted between October and November 2021.

### Study instrument

A 33-itemized semi-structured questionnaire comprising three sections was applied by a face-to-face interview and used for data collection (available as supplementary material 1 in Spanish, the original language of application. In addition to the nine questions on demographic data, 10 questions explored knowledge of rabies, and 14 questions focused on attitudes and practices towards the disease. Respondents were asked to answer in limited and multiple-choice formats. The primary version of the knowledge, attitudes, and practices survey was adapted from similar studies carried out elsewhere ^9^^,^^14^^,^^15^.

The questionnaire was pre-tested at a small scale to evaluate its effectiveness, acceptability, and consistency. Three experts in the field assessed the structure to ensure that all important issues were identified and covered, and to identify problems such as unnecessary length, poorly worded, unclear questions, or allowance of subjective responses. In addition, the questionnaire was applied to a small proportion of pet owners (n = 5), seeking to identify interpretation problems ^16^. Minor modifications were needed after the pilot *(i.e.,* modifications to some of the technical terms, clarity in one question). Data derived was not included in the final analysis.

As the consistency and validity of the study questionnaire were defined ^17^, the instrument was available for data collection. It was applied to the study population by veterinary staff previously trained for the task through instruction meetings and simulacrums with peers. In addition, direct monitoring was carried out by the researchers to the interview and information collection processes.

The Google Forms tool was used, and data was transferred from tablets to a server and compiled into an Excel^®^ spreadsheet (Microsoft Corp., Redmond, WA, USA). A knowledge, attitudes, and practices work plan (available as supplementary material 2 in Spanish, original language of the application, was also built to monitor compliance with the steps defined by the *a priori* strategy.

### Data analysis

Descriptive statistics were calculated for all variables. The median (50^th^ percentile) was calculated only to define categories for the variable 'age', and primary and non-formal categories were compiled into basic for the variable 'educational level'. Multiple correspondence analysis was used as an exploratory method to summarize and describe the information of categorical variables. In this case, data from a knowledge, attitudes, and practices survey was classified into two dimensions (knowledge and attitudes, and practices), simplifying a complex data set composed of qualitative variables 13,18,19. All the dimensions extracted from the multiple correspondence analysis were explored, and the one with higher inertia was chosen for the analysis.

The essential analysis steps included determining the number of dimensions, excluding variables with floor or ceiling effects, and analyzing groups of variables related to knowledge, attitudes, and practices. Additional demographic variables, such as education, were incorporated to understand the factors influencing knowledge, attitudes, and practices.

An exploratory analysis, using hierarchical clustering, was performed to identify participant groups with similar response patterns. The "biplot" technique visualized relationships between variables and participant groups.

Although detailed knowledge, attitudes, and practice profiles were not identified due to response heterogeneity, the results did show trends. 'Educational level' was selected as the indicator variable based on its relevance and strong associations with other variables. This approach ensured a comprehensive understanding of knowledge, attitudes, and practice profiles while avoiding technical details of the multiple correspondence analysis ^20^. The 'educational level' was defined as the indicator variable for the definite multiple correspondence analysis analysis, according to the results obtained. Graphical results obtained after exploring three other potential variables (age, binary gender, and place of work) are available as supplementary material 3.

The idea behind selecting the indicator variable was to use a combination of statistical analysis, expertise, and plausibility to identify a variable that could effectively capture and represent the dimensions of knowledge, attitudes, and practices in the study. Multiple correspondence analyses were performed with the FactoMineR package of R^®^ software, version. 4.1.1, 2021 (R Foundation for Statistical Computing, Vienna, Austria).

Heatmap tables facilitated a clear visual representation of the collected data, charting the perceptions of the survey participants with a color-coding scheme ranging from intense red (0%) to intense green (100%). Each cell in the corresponding table represents a specific response related to perceptions towards rabies, and the assigned color indicates the percentage of participants sharing that perception. Employing this methodology allowed us to identify significant patterns and areas of greater relevance in the population's perceptions regarding rabies and to strengthen the epidemiological understanding of this public health issue.

### Ethical considerations

The *Comité de Ética y Bioética para Experimentación Animal* of the *Universidad Cooperativa de Colombia* approved this study (Bioethical Concept No. BIO13, Act No.004 of November 4^th^, 2021). Written informed consent was also considered, including a confidentiality guarantee from the respondents before starting the interview.

## Results

The population of the study was domiciled in Pan de Azúcar (20/71; 28.2%), La Martinica (19/71; 26.8%), Cerro Gordo (17/71; 23.9%), and Noroccidentales (15/71; 21.1%) tutelary hills, and the residence areas were mainly urban.

A significant proportion of the participants resided in urban areas (67.6%). The median age was 46 (IQR = 31.5 - 57.5), and 74.6% were women. Most participants had a middle (high school: 46%) or basic education (primary and non-formal: 35%), while a smaller proportion had a higher education (graduate: 18%). A significant majority worked in the city (90%).

Most households had one (54.9%) or two (28.2%) inhabitants. The number of children under 14 years varied per household, with 50.7% not having and the remaining participants having 1-5 children, with decreasing percentages as the number of children increased.

Most subjects had one dog (50.7%), while smaller percentages had 2-7 dogs, with the proportion decreasing as the number of dogs increased. Similarly, most households had one cat (45.1%), with a smaller percentage having 2-11 cats. The characterization of the 71 pet owners included in the study is in table 1.

**Table 1:** Sociodemographic characteristics of the 71 pet owners included in the study

Item		Frequency	Distribution
		(n)	(%)
Residence area			
	Urban	48	67.6
	Rural	23	32.4
Age (years)*			
	< 46	35	49.3
	≥ 46	36	50.7
Binary gender*			
	Female	53	74.6
	Male	18	25.4
Educational level*			
	Basic (primary and non-formal)	25	35.2
	Middle (high school)	33	46.5
	High (graduate)	13	18.3
Place of work*			
	In the city	64	90.1
	In rural areas	7	9.9
Number of inhabitants per household**			
	1	20	28.2
	2	39	54.9
	3	12	16.9
Number of children (< 14 years old)			
	0	36	50.7
	1	14	19.7
	2	12	16.9
	3	6	8.5
	4	2	2.8
	5	1	1.4
Number of dogs per household			
	1	37	52.1
	2	18	25.4
	3	8	11.3
	4 or more	8	11.2
Number of cats per household			
	1	35	49.3
	2	23	32.4
	3	6	8.5
	4 or more	7	9.8

* Information of the respondent

** Including the respondent

The functioning profile of dimension 1 (knowledge) stratified by demographic characteristics of the 71 pet owners of the study is shown in table 2. All respondents correctly identified the ways in which the disease can be contracted by humans (bites, scratches, licking, airborne transmission). Therefore, the related question was not included in the data analysis. We observed a high level of recognition regarding the possibility of contracting rabies, with percentages ranging from 77.1 to 100% across different demographic groups. This knowledge is more prominent among individuals residing in rural areas (91.3%), those aged 46 years or older (94.4%), males (88.9%), individuals with higher education (84.9%), and city-based workers (90.1%).

**Table 2:** Heatmap for the dimension 1 (knowledge) stratified by demographic characteristics of the 71 pet owners included in the study.

Question (n = 71; 100%)	Residence area		Age (years)		Binary gender		Educational level			Place ofwork	
	Rural	Urban	< 46	≥ 46	Male	Female	Basic	Middle	High	Rural	City
	23 (32.4%)	48 (67.6%)	46 (31.5-57.5)*		18 (25.3%)	53 (74.7%)	25 (35.2%)	33 (46.5%)	13(18.3%)	7 (9.9%)	64 (90.1%)
Recognizes that a person can get rabies.	91.3%	83.3%	77.1%	94.4%	88.9%	84.9%	84%	81.8%	100%	100%	84.4%
Recognizes that rabies can be fatal to humans.	73.9%	64.6%	65.7%	69.4%	61.1%	69.8%	68%	60.6%	84.6%	85.7%	65.6%
Recognizes that rabies is a preventable disease in humans.	87%	87.5%	82.9%	91.7%	88.9%	86.8%	80%	87.9%	100%	100%	85.9%
Recognizes the nature of rabies etiological agent.	82.6%	22.9%	22.9%	25%	16.7%	26.4%	24%	18.2%	38.5%	14.3%	25%
Recognizes that animals can transmit rabies and infect a person.	82.6%	79.2%	88.6%	72.2%	83.3%	79.2%	68%	81.8%	84.6%	100%	78.1%
Identifies how a person can get rabies from an animal.	100%	100%	100%	100%	100%	100%	100%	100%	100%	100%	100%
Identifies what animals can become infected with rabies**.	87%	72.9%	82.9%	72.2%	88.9%	73.6%	64%	87.9%	92.3%	71.4%	78.1%
Identifies the symptoms exhibited by an RABV-infected animal***.	13%	35.4%	17.1%	38.9%	27.8%	28.3%	28%	24.2%	38.5%	14.3%	29.7%
Knows that a friendly dog or cat that suddenly becomes aggressive may have rabies. Knows that if a bat flies during the day, awkwardly, and crushing with objects and walls (erratic flight), can have rabies.	56.5%	56.3%	57.1%	55.6%	38.9%	62.3%	76%	84.8%	61.5%	57.1%	56.3%
	52.2%	39.6%	37.1%	50%	44.4%	43.4%	40%	45.5%	46.2%	42.9%	43.8%

* Median (IQR)

** Identifies at least two animal species, even if they are terminal hosts (i.e. bat, dog, cat, fox, cow, horse, pig) *** Identifies at least two symptoms (i.e. fever, hoarse barking, aggression, photophobia, hydrophobia, sialorrhea).

### Heat scale (%)

**Table:** 

0%	10%	20%	30%	40%	50%	60%	70%	80%	90%	100%

Furthermore, understanding that rabies can be fatal to humans also exhibited significant levels, ranging from 64.6 to 85.7%. In this context, individuals living in rural areas (73.9%), those younger than 46 years (65.7%), women (69.8%), individuals with basic education (68%), and field workers (78.1%) demonstrated slightly lower percentages.

On the other hand, there is a general awareness that rabies is preventable in humans, with percentages varying from 80 to 100%. Notably, higher levels of awareness are observed among individuals residing in rural areas (87%), those aged 46 years or older (91.7%), males (88.9%), individuals with higher education (86.8%), and city-based workers (100%).

Regarding the recognition of the etiological agent of rabies, the percentages were generally lower across all demographic characteristics, with values ranging from 14.3 to 26.4%, except for individuals coming from rural areas, where the recognition was notably higher (82.6%). Among individuals living in urban areas, the proportion was 22.9%, while for those aged 46 years or older, it was 25%. These findings highlight the need for targeted educational campaigns to improve awareness of the etiological agent of rabies, particularly among urban residents and older age groups.

The analysis further emphasizes that the knowledge about how a person can contract rabies from an animal is universally recognized, with 100% observed across all demographic groups, indicating a high level of awareness in this aspect. However, symptom identification in the infected animals was considerably low, particularly among individuals residing in rural areas, where only 13% demonstrated awareness of symptoms.

The functioning profile of dimension 2 (attitudes and practices) stratified by demographic characteristics of the 71 pet owners of the study is shown in table 3. A significant percentage of participants residing in rural areas allowed their pets to wander alone outside their homes (73.9%) or permitted stray dogs and cats to enter their houses (82.6%). In addition, cases of animal bites in the past were identified, with 26.1% occurring in the last month, emphasizing the importance of appropriately addressing such incidents to prevent rabies transmission.

**Table 3:** Heatmap for dimension 2 (attitudes and practices) stratified by demographic characteristics of the 71 pet owners included in the study

Question (n = 71; 100%)		Residence area		Age (years)		Binary gender		Educational level			Place ofwork	
		Rural	Urban	< 46	≥ 46	Male	Female	Basic	Middle	High	Rural	City
		23 (32.4%)	48 (67.6%)	46 (31-57)*		18 (25.3%)	53 (74.7%)	25 (35.2%)	33 (46.5%)	13(18.3%)	7 (9.9%)	64 (90.1%)
Allows pets to wander alone outside the household.		73.9%	41.7%	51.4%	52.8%	38.9%	56.6%	68%	45.5%	38.5%	71.4%	50%
Allows stray dogs or cats to enter their homes.		82.6%	70.8%	77.1%	72.2%	77.8%	73.6%	72%	75.8%	76.9%	85.7%	73.4%
Catches and helps bats found on the floor.		43.5%	29.2%	34.3%	33.3%	27.8%	35.8%	28%	33.3%	46.2%	28.6%	34.4%
Has been bitten by a dog, cat, or bat (the bite does not have to be serious).	In the last month	26.1%	2.1%	11.4%	8.3%	11.1%	9.4%	4%	15.2%	7.7%	14.3%	9.4%
	In the last 2 to 6 months	4.3%	2.1%	5.7%	0%	0%	3.8%	0%	6.1%	0%	0%	3.1%
	More than 6 months ago	34.8%	52.1%	45.7%	47.2%	50%	45.3%	36%	45.5%	69.2%	28.6%	48.4%
	Never	34.8%	43.8%	37.1%	44.4%	38.9%	41.5%	60%	33.3%	23.1%	57.1%	39.1%
Identifies the correct immediate actions with the animal when it has bitten a person**.		30.4%	22.9%	31.4%	19.4%	11.1%	30.2%	12%	24.2%	53.8%	30.4%	22.9%
Recognizes the importance of washing the wound immediately with soap and water after a bite.		95.7%	91.7%	94.3%	91.7%	88.9%	94.3%	96%	87.9%	100%	100%	92.2%
Identifies the correct immediate actions when an animal show rabies-related symptoms.		82.6%	89.6%	82.9%	91.7%	83.3%	88.7%	84%	87.9%	92.3%	85.7%	87.5%
Identifies the correct immediate actions when an animal presenting rabies symptoms has died.		0%	6.3%	0%	8.3%	5.6%	3.8%	12%	0%	0%	0%	4.7%
Recognizes the importance of vaccination of dogs and cats to prevent rabies in humans.		95.7%	91.7%	88.6%	97.2%	88.9%	94.3%	96%	87.9%	100%	100%	92.2%
Identifies the frequency of vaccination of dogs and cats against rabies		39.1%	39.6%	34.3%	44.4%	38.9%	39.6%	36%	39.4%	46.2%	42.9%	39.1%
Self-appraisal of the knowledge about the disease	Enough	0%	29.2%	14.3%	25%	33.3%	15.1%	24%	18.2%	15.4%	0%	21.9%
	Scarce	87%	62.5%	71.4%	69.4%	50%	77.4%	68%	69.7%	76.9%	100%	67.2%
	None	13%	8.3%	14.3%	5.6%	16.7%	7.5%	8%	12.1%	7.7%	0%	10.9%
	Interested in more information about the disease	100%	93.8%	97.1%	94.4%	100%	94.3%	92%	97%	100%	100%	95.3%

* Median (IQR)

** Compiles responses to two questions

*** According to the National Rabies Program of Colombia

### Heat scale (%)

**Table:** 

0%	10%	20%	30%	40%	50%	60%	70%	80%	90%	100%

Regarding immediate actions following an animal bite, approximately 95.7% of rural participants and 100% of those with a higher educational level recognized the importance of washing the wound with soap and water. However, only 30.4% identified the correct actions to take with the animal in case of a bite. Notably, although participants had a high level of awareness about the significance of vaccinating dogs and cats to prevent rabies in humans (95.7%), only 39.1% of the participants knew about the frequency of pet vaccination against rabies.

More urban (67.6%) than rural (32.4%) participants recognized that a person could contract rabies. Moreover, 70.8% of urban respondents allowed stray dogs and cats to enter their homes, in contrast to 82.6% of rural participants. This fact could be linked to the varying exposure to stray animals in urban and rural settings.

Regarding age, participants over 46 (91.7%) exhibited greater awareness of the importance of immediately washing the wound after a bite compared to those under 46 (82.9%). This evidence suggests that accumulated experience and knowledge can influence preventive practices against rabies.

More women (74.7%) than men (25.3%) recognized that a person can contract rabies. However, more men (44.4%) than women (39.6%) knew about the frequency of vaccination of dogs and cats against rabies.

Participants with secondary education (46.5%) and higher (18.3%) were more aware that a person can contract rabies compared to those with basic education (35.2%). Furthermore, those (100%) with higher education completely understood the importance of vaccinating dogs and cats to prevent rabies, compared to 87.9% of participants with secondary education and 92.2% with basic education.

Less individuals working in rural areas (9.9%) than those in urban settings (90.1%) recognized that a person can contract rabies. In addition, 76% of participants working in urban areas correctly identified the immediate actions to take when an animal shows symptoms of rabies, compared to 68% of those working in rural areas.

**Figure 1:**
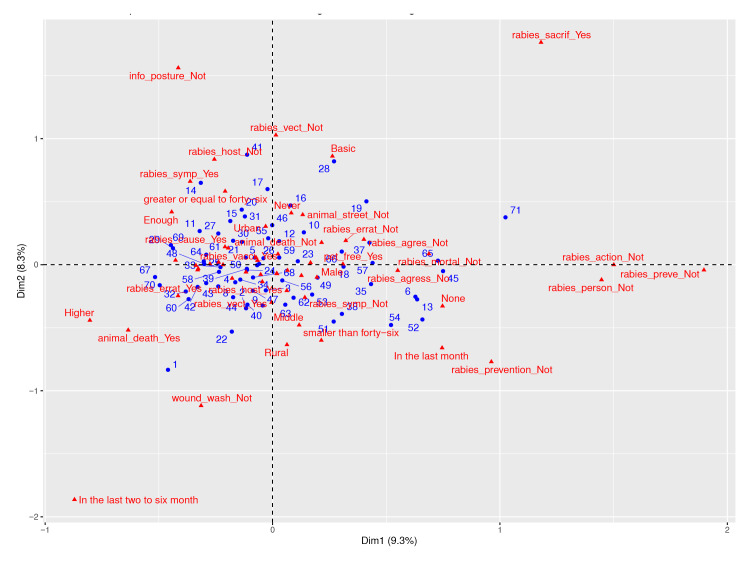
Simultaneous representation of respondents and categories of the two domains (i.e. knowledge, attitudes, and practices)

Data analysis revealed two dimensions explaining 17.6% of the variance: dimension 1 (knowledge) explained 9.3%, and dimension 2 (attitudes and practices) explained 8.3%. In dimension 1, respondents' knowledge about rabies showed variations across educational levels, while dimension 2 captured attitudes and practices related to the disease. We found strong associations between respondents lacking knowledge about handling animal bites and the belief in euthanizing suspected infected animals. A significant link was also observed between the lack of knowledge of preventive measures and appropriate actions when faced with symptomatic animals. Figure 1 provides valuable insights into these variations and deficiencies.

## Discussion

The present research approach aimed to determine knowledge, attitudes, and practice profiles ^21^ regarding rabies in a municipality in Colombia, using the multiple correspondence analysis technique. The motivation behind this study lies in the Colombian epidemiological status, where rabies remains endemic. By analyzing the knowledge, attitudes, and practices in the municipality of study, we aimed to gain insights into the specific context of rabies-related perceptions to identify strategic areas for intervention and improvement in disease prevention and control. Our findings could contribute to proposing more effective public health policies and programs targeting rabies in Colombia, addressing the unique challenges of this disease in the country.

In Colombia, national programs focused on mass dog vaccination have reduced the instances of dog-transmitted rabies, with genetic lineage circulation limited to a few municipalities in the national territory. Since February 2007, urban rabies has notably decreased ^22^. However, sylvatic rabies has emerged as a growing threat, now causing most of the rabies-related mortality in humans and production animals.

On the other hand, due to their proximity to humans and varied hunting habits that include bats, cats have become the most efficient intermediate vector in the transmission of bat rabies to humans ^23^. Since 2012, cats have been the sole intermediary responsible for rabies transmission to humans, with cases occurring during this period in the departments of Valle (Roldanillo), Cundinamarca (El Colegio, Girardot, and Tena), and Huila (Neiva and La Argentina).

It is well known that human deaths due to RABV are 100% preventable through immediate methods, such as washing the bite with plenty of water and soap, immediately administering post-exposure prophylaxis in people bitten by suspicious and potential rabies-transmitting animals, and extensive vaccination campaigns for domestic animals ^24^. As an additional aspect to consider, people need to be aware of the risks associated with rabies and the necessary actions to prevent human infection, which translates into knowledge about the disease and improvement in practices for disease control and prevention ^14^.

This reality is not alien to Colombia, where, despite the existence of a vaccine and ongoing epidemiological surveillance from government entities, rabies cases continue to occur: 44 fatal cases in humans reported to date ^25^. As a unique feature, within Colombian territory, cats play a significant role as species that attack and transmit, contributing to virus maintenance. In this sense, it is crucial to emphasize the importance of continuous epidemiological surveillance and the need for comprehensive strategies to control and prevent rabies in the country, considering the specific challenges posed by the involvement of cats in transmission ^26^.

Efforts should focus on promoting responsible pet ownership, increasing vaccination coverage, and implementing effective control measures to reduce the incidence of rabies in the country while aligning with global strategies ^27^.

These proactive actions have the potential to yield significant positive effects on the overall outcome of the programs. Promoting responsible pet ownership, such as ensuring proper confinement and care, can significantly reduce the risk of rabies transmission. Increasing vaccination coverage among dogs and cats in urban and rural areas is crucial for achieving herd immunity and preventing the spread of the disease. Effective control measures, such as surveillance and monitoring systems, can help identify and respond to potential outbreaks promptly. Additionally, education and awareness campaigns can play a vital role in informing the public about the importance of rabies prevention, proper handling of pets, and early recognition of symptoms. By addressing these aspects comprehensively, the programs can not only reduce the incidence of rabies but also contribute to the overall improvement of public health and safety in the country.

The assessment of knowledge, attitudes, and practices through surveys has proven to be a valuable tool in understanding infectious diseases of zoonotic interest worldwide. Authors in Tanzania ^14^, India ^15^, Brazil ^28^, Indonesia ^29^, Cambodia ^30^, Thailand ^31^r, Mozambique ^32^, India ^33^, Uganda ^34^, and Pakistan ^9^^,^^35^have contributed to this body of research in rabies. Building upon their work, we developed our starting point to assess knowledge, attitudes, and practice profiles within the population of interest.

Only two studies have been conducted in Colombia on the population's perception about zoonotic diseases. One study focused on identifying the major zoonoses in Bogotá ^36^, and the second examined zoonoses among the general population in a rural area in the central region of the country ^37^.

Utilizing knowledge, attitudes, and practices surveys in the context of an infectious disease, such as rabies, offers advantages and limitations. This kind of survey provides valuable insights into the target population, aiding in the identification of risk factors and misconceptions associated with the disease. This design facilitates the development of targeted interventions and educational programs to address knowledge gaps and promote positive behavioral changes.

However, the reliance on self-reported data introduces potential biases and socially desirable responses, affecting the accuracy of the findings. In addition, the generalizability of results may be limited due to specific population targeting and sample size considerations. Despite these limitations, leveraging the strengths of knowledge, attitudes, and practices surveys can enhance our understanding of infectious diseases like rabies and inform public health interventions for improved prevention and control.

Due to the clustering of variables, we did not identify clear profiles in the present study. The associations between variables were not different, making it challenging to identify distinct groups of respondents based on their characteristics. Nevertheless, the analysis provided valuable insights into respondents' knowledge, attitudes, and practices related to rabies across different educational levels, emphasizing the need for tailored educational strategies to address misconceptions and improve overall awareness and behavior regarding rabies prevention and control.

The multiple correspondence analysis approach -as an exploratory data analysis technique- allows the examination of complex patterns and relationships among multiple categorical variables. Its application in this context provides a detailed understanding of how the investigated variables are related and how they may influence the respondents' answers. Its use in epidemiological studies related to infectious diseases, such as rabies, is essential for understanding the underlying factors that influence the disease's spread and the population's responses to it ^38^^,^^39^. By exploring the associations between knowledge, attitudes, and practices and participant characteristics, a more comprehensive picture of the current situation in terms of rabies-related outcomes in the population of study could be obtained.

The results obtained through multiple correspondence analyses revealed significant associations between educational level and respondents' answers regarding rabies. This indicates that that educational level playsa crucial role in the acquisition and understanding the necessary knowledge to prevent and manage the disease. These findings are consistent with previous studies highlighting the importance of health education to improve prevention and control of infectious diseases ^40^. Our results also revealed significant differences in knowledge, attitudes, and practices according to educational level. The responses of individuals with a higher educational level reflected greater alignment with their educational background. In contrast, those with a basic education lacked adequate knowledge and attitudes regarding rabies.

Dimension 1 (knowledge) of our study focused on exploring participants' knowledge regarding rabies prevention and appropriate actions when encountering an animal with symptoms compatible with the disease. A strong association was found between the lack of knowledge about rabies prevention and the lack of knowledge about what to do when encountering such an animal. This finding suggests the need for specific educational programs aimed at improving knowledge about rabies prevention and proper management among different population groups; also, it highlights the importance of addressing knowledge gaps and negative attitudes towards the disease, particularly among groups with lower levels of education, through tailored education and awareness programs that cater to their specific needs. Such lack of knowledge is evident from the majority response of "Don't know" to questions related to the causal agent and symptoms of rabies.

Other studies reported similar findings when examining rabies knowledge in different populations. They identified a significant percentage of participants unaware of the cause of rabies ^41^ and with limited familiarity with the symptoms exhibited by infected animals ^42^. Similar trends in knowledge have been reported in studies conducted in Kenya, Tanzania, and Guatemala, with knowledge rates of 49, 27, and 0%, respectively ^43^. These findings support and reinforce that the lack of knowledge about rabies is a common problem in different contexts and populations.

Nevertheless, some respondents demonstrated a basic understanding of the disease. For example, a significant proportion recognized that rabies can be fatal to humans and that animals can transmit the disease to humans. These findings are consistent with results from other studies that have emphasized the importance of raising awareness about the severity of rabies and the need to avoid contact with potentially infected animals ^34^.

John Snow -a pioneering figure in epidemiology- once stated that understanding the spread patterns of infectious diseases requires careful examination of both individual cases and their broader context and can be more effective in controlling the disease than even knowing the or about the causative agent ^44^.

In line with Snow's premise, our study sheds light on the importance of attitudes and practices about rabies in the prevention and control of the disease, even over results about dimension 1 (knowledge). In this sense, we observed a significant association between educational level and dimension 2 (attitudes and practices) regarding rabies prevention. For example, allowing pets to wander alone outside the household is a common practice in all categories, with a decreasing tendency as the socioeconomic level increases. This situation indicates that people from higher socioeconomic categories tend to have better control over their pets, preventing them from freely roaming the neighborhood. In addition, such individuals showed a greater tendency to properly vaccinate their pets and take preventive measures, such as contacting the relevant authorities when suspecting an animal with rabies-compatible symptoms.

On the other hand, those with a basic educational level lacked knowledge in identifying signs of rabies in animals and perceiving the associated risk with bat behavior. These findings highlight the value of education and health promotion in rabies prevention ^45^.

It is important to note that the associations found in this study are influenced by various socioeconomic and cultural factors that may interact with the educational level. Previous studies have indicated that factors such as access to healthcare services, geographical location, and cultural beliefs on attitudes and practices could influence rabies prevention ^46^. Therefore, it is essential to consider these contextual factors when designing educational interventions and raising awareness about the disease.

Another relevant aspect is the association between a lack of knowledge about rabies prevention and what to do when facing an animal with symptoms compatible with the disease. Individuals who did not demonstrate awareness in both areas lacked the knowledge and attitudes to handle rabies appropriately. This situation underscores the importance of providing accurate and accessible information about rabies prevention and management, especially for those with a basic educational level (primary and non-formal). These findings are consistent with previous research that has emphasized the importance of education in health promotion and disease prevention. Education has been previously reported as a factor that can increase the understanding of the disease, promote positive attitudes toward preventive measures, and foster healthy behaviors related to rabies ^47^. Therefore, it is crucial to develop comprehensive educational programs that address the identified gaps in this study.

In addition, we found a strong association between a lack of knowledge about what to do when a dog or cat bites a person and the belief that animals should be euthanized in response to aggression. This association indicates a lack of knowledge about proper animal handling and control measures after a bite, and a potential attitude of fear or stigmatization towards aggressive animals. Previous research has highlighted the importance of adopting evidence-based approaches to animal bite management and avoiding unnecessary euthanasia of healthy animals ^48^.

The results of this study emphasize the need to develop specific educational strategies targeted at different segments of the population, considering the education level and socio-cultural features. These strategies may include public awareness campaigns, programs in schools, and training for healthcare professionals and veterinarians to improve knowledge about rabies, foster positive attitudes toward prevention, and promote appropriate practices in animal handling and bite prevention.

Human activity has encroached upon natural spaces, altering the dynamics of interaction with wildlife. Bats, which play a role in rabies transmission, further complicate the disease. There is generally a negative perception of bats among people, which has also been subject of study. It is important to address this perception and promote a more comprehensive understanding of rabies to encourage effective prevention and control measures ^49^.

This study has several strengths. To the authors' knowledge, this approach stands out as the first one applying multiple correspondence analysis to data about knowledge, attitudes, and practices towards rabies in Colombia. This fact is relevant because it provides direct and up-to-date information on people's perceptions and behaviors related to this disease. In addition, it is worth highlighting that multiple correspondence analysis allowed an in-depth exploration of the relationships between variables, facilitating the identification of significant patterns within the survey data. This innovative application of multiple correspondence analysis in the context of rabies in the surveyed population offers a more comprehensive and detailed insight into knowledge, attitudes, and practices related to this zoonosis. Under the One Health approach, the design and analysis of knowledge, attitudes, and practice surveys provide relevant data regarding the perception and behaviors of the surveyed individuals and the rabies-transmitted animals. This focus provides a holistic view of the situation and allows identification of potential intervention points and improvement in the prevention and control of the disease.

This study faced budget limitations, compromising the research scope and data collection strategies. Consequently, the sample size, geographic coverage, and depth of data analysis may have been affected. These limitations can result in a lack of representativeness and proportionality of the sample, as well as a non-multistage design due to a small sample size, potentially impacting the generalizability of the results. Other limitations were related to security concerns in the study area, which prevented access to some remote locations. Therefore, it is important to interpret the results cautiously and acknowledge the need for larger and more representative studies considering funding and methodological constraints. Future studies should aim to capture the diversity of opinions and attitudes within the target population and provide a more comprehensive understanding of rabies-related practices. Furthermore, addressing security concerns in the study area would facilitate broader data collection and enhance the effectiveness of prevention and control strategies.

On the other hand, while it was not an objective of the current study -and thus, no related questions were included in the questionnaire-, other important variables for determining rabies risk approaches include bat presence in the area and history of outbreaks in livestock. It is also important to review potential deficiencies in the national control program or insufficient intervention in the area, despite the actions established by the *Instituto Nacional de Salud* regarding rabies in dogs and cats and the *Instituto Colombiano Agropecuario* in livestock. Despite such limitations, this study provides an initial foundation for future, broader, and more comprehensive research of attitudes and practices related to rabies with the interest of facilitating the implementation of more effective prevention and control strategies.

Limitations can be overcome using cluster sampling designs to achieve greater coverage and representativeness of the target population. Furthermore, larger sample sizes will ensure better statistical precision and higher participant diversity. These methodological improvements will strengthen the validity and reliability of studies on knowledge, attitudes, and practices regarding rabies in the Colombian territory.

In conclusion, this study represents a significant advancement in understanding attitudes and practices of rabies prevention in the surveyed population. It also serves as the first "face-to-face" approach with communities, instilling a sense of motivation to actively participate in controlling this deadly disease. The results highlight the importance of health education and the implementation of evidence-based prevention strategies. In this sense, it is crucial to design educational and awareness programs that address the identified gaps in the three investigated dimensions, especially among those with higher levels of education. In addition, it is essential to consider sociocultural and contextual factors to promote effective prevention practices within the community. Furthermore, the importance of the One Health approach in the prevention and control of zoonotic diseases, such as rabies, is emphasized as it addresses the interconnectedness of human, animal, and environmental health. These collaborative efforts will contribute to reducing rabies incidence and improve Colombian population health, thus aligning with the global strategy of "Zero by 30".

## Suplemmentary material

**Supplementary material 1:** Knowledge, attitudes, and practices survey in Spanish

Estimado tutor:					
Deseamos conocer sus **CONOCIMIENTOS, ACTITUDES Y PRÁCTICAS (CAP)** para obtener información acerca de (1 ) el conocimiento que tiene de la rabia, su transmisión y resultado, especies afectadas y medidas de prevención y control, y (2) actitudes y prácticas orientadas a la prevención de la enfermedad y manejo de los animales y cadáveres sospechosos de rabia. La información que proporcione se utilizará para mejorar el control de la enfermedad en el país. Igualmente, su participación es voluntaria y puede optar por detener la encuesta en cualquier momento y se garantiza su anonimato y la completa confidencialidad de la información suministrada.					
Gracias por su tiempo.					
INFORMACION DE LA ENCUESTA Y GEORREFERENCIACION					
ID (consecutivo) de perro y gato muestreados en el domicilio					
Fecha de la encuesta					
Nombre del encuestador					
Datos de georreferenciación					
Longitud		Latitud		Altitud	
PREGUNTAS GENERALES Y SOCIODEMOGRAFICAS					
PREGUNTA			RESPUESTAS	PUNTUACION	CATEGORIZACION
1. Zona de residencia			Urbano		
			Rural		
2. Edad (en años)					
3. Género			Masculino		
			Femenino		
4. Nivel de educación			No formal		
			Básica (primaria)		
			Media (bachillerato)		
			Superior (universitario)		
5. Ocupación			Labores en ciudad		
			Labores en campo		
6. Número de personas que habitan en el domicilio, incluyendo al entrevistado					
7. Número de niños menores de 14 años, que habitan en el domicilio					
8. Número de perros que habitan en el domicilio					
9. Número de gatos que habitan en el domicilio					
CONOCIMIENTOS Y CONCIENCIA FRENTE A LA RABIA					
10. ¿Qué cree usted que causa la enfermedad de la rabia?			Un virus		
			Una bacteria		
			Un parásito		
			Otro ¿cuál?		
			NOSE		
11. ¿Cuáles animales pueden enfermarse de rabia?			Murciélagos		
			Perros		
			Gatos		
			Zorros		
			Vacas		
			Caballos		
			Cerdos		
			Aves		
			Reptiles (por ejemplo, iguanas)		
			Otros ¿cuáles?		
12. ¿Cree usted que una persona puede enfermarse de rabia?			SI		
			NO		
13. ¿Cree usted que la rabia puede ser mortal para las personas?			SI		
			NO		
14. ¿La rabia es una enfermedad que se puede prevenir en las personas?			SI		
			NO		
15. ¿Qué animales pueden contagiar a una persona de rabia? (seleccione el número de opciones necesarias)			Perro		
			Gato		
			Zorros		
			Zarigüeya ("chucha")		
			Murciélago		
			Vaca		
			Caballo		
			Cerdo		
			Pájaros		
			Reptiles (iguana)		
			Otros ¿cuáles?		
16. ¿Cómo puede una persona contraer la rabia? (seleccione el número de opciones necesarias)			Mordedura		
			Arañazo		
			Lamido		
			Vía aérea		
			Las personas no pueden contraer la rabia		
			NO SÉ		
17. ¿Cuáles son los síntomas que presenta un animal con rabia? (seleccione el número de opciones necesarias)			No puede tomar agua		
			Se le cae el pelo		
			Fiebre		
			Ladrido ronco		
			Babeo-salivación excesiva		
			Muerde/ataca cualquier objeto		
			Se esconde de la luz		
			No hace nada/no presenta cambios		
			NOSE		
18. Un perro o gato que es amistoso y de repente se vuelve agresivo, ¿puede tener rabia?			SI		
			NO		
19. Si un murciélago vuela de día, torpemente y chocándose con los objetos y paredes (vuelo errático), ¿puede tener rabia?			SI		
			NO		
ACTITUDES Y PRACTICAS FRENTE A LA RABIA					
20. ¿Deja que su mascota (perro gato) deambule sola por fuera del domicilio			SI		
			NO		
			No me doy cuenta si sale		
21. ¿Cuál considera usted que debe ser la frecuencia de vacunación de perros y gatos contra la rabia?			Cada 6 meses		
			Cada año		
			Cada *2a3* años		
			Una vez en la vida		
			La vacuna no es necesaria		
22. Si encuentra un perro o gato en situación de calle ¿le da ingreso a su casa para ayudarlo?			SI		
			NO		
			Amplíe su respuesta		
23. Si encuentra un murciélago en el piso, ¿lo atrapa y lo llevo a otro lugar para ayudarlo?			SI		
			NO		
			Amplíe su respuesta		
24. Si ve un animal (murciélago, perro, gato) con sintomatologia correspondiente a rabia (mencionada antes), ¿qué considera que debe hacer inmediatamente?			Comunicarse con la Secretaría de salud municipal		
			Llamar al veterinario de confianza		
			Comunicarse con la policía del sector		
			Llevarlo a su casa y tenerlo en observación		
			No debo hacer nada		
			No sabría lo que debo hacer		
25. Si un animal presenta sintomatologia compatible con rabia y este muere, ¿qué debo hacer inmediatamente?			Enterrar al cuerpo del animal		
			Llamar a las entidades de salud correspondientes		
			Llamar a un veterinario		
			No debo hacer nada		
			No sabría lo que debo hacer		
26. ¿Ha sido mordido por un perro, gato o murciélago? (la mordedura no tiene que ser necesariamente grave)			Nunca		
			En el último mes		
			En los últimos *2a6* meses		
			Hace más de 6 meses		
27. Si un perro o gato muerde a una persona, ¿el animal debería ser sacrificado inmediatamente?			SI		
			NO		
			SI		
28. Si un perro o gato muerde a una persona, ¿considera usted que es una buena medida lavarse la herida con agua y jabón de manera inmediata, con el fin de evitar contagiarse de rabia?			NO		
29. ¿Qué se debe hacer cuando un perro o gato muerde a una persona? (incluso si la lesión no es grave)			Ir a la farmacia y preguntar por un tratamiento		
			Tratar con medicina tradicional (por ejemplo, hierbas, pomadas)		
			Ir al centro de salud u hospital más cercano		
			No debe hacer nada		
			No sabría lo que debo hacer		
30. ¿Considera usted que la rabia en humanos se puede prevenir al vacunar perros y gatos contra la enfermedad?			SI		
			NO		
31. ¿En cuanto a la información que tiene de la enfermedad considera que?			Es suficiente		
			Es poca		
			No tiene		
			Otra respuesta		
32. ¿Le gustaría poder tener más información acerca de esta enfermedad?			SI		
			NO		
33. ¿Cuáles son las fuentes de información que cree que pueden llegar de manera más efectiva a personas como usted con información sobre la rabia? (elija las tres fuentes más eficaces).			Periódicos y revistas		
			Radio		
			Televisión		
			Vallas publicitarias		
			Folletos, carteles y otros materiales impresos		
			Trabajadores de la salud		
			Líderes comunitarios		
			Otro ¿Cuál?		

**Supplementary material 2:** Knowledge, attitudes, and practices workplan

ACTIVIDAD		2021								2022	2023
MAYO	JUNIO	JULIO	AGOSTO	SEPTIEMBRE	OCTUBRE	NOVIEMBRE	DICIEMBRE		
1	DEFINICION DE LOS OBJETIVOS DE LA ENCUESTA										
1.1	Revisión de los materiales existentes (literatura)										
T.2	Determinación del propósito de la encuesta										
1.3	Identificación de las áreas de investigación										
1.4	Identificación de la población objeto de la encuesta										
Ts	Creación del plan de muestreo										
2	DESARROLLO DEL PROTOCOLO DE LA ENCUESTA										
2.1	Organización de los contenidos del protocolo										
2.2	Identificación de las preguntas de investigación claves										
2.3	Determinación de la necesidad de una revisión ética										
2.4	Creación del plan de muestreo										
2.5	Desarrollo del presupuesto										
3	DISEÑO DEL CUESTIONARIO DE LA ENCUESTA										
3.1	Desarrollar el cuestionario de la encuesta										
3.2	Realicación de prueba piloto y definición de la encuesta definitiva										
3.3	Definición del plan de análisis de datos										
3.4	Elección de las fechas de la encuesta										
4	REALIZACION DE LA ENCUESTA TIPO KAP										
4.1	Reclutamiento y capacitación de supervisores y entrevistadores										
4.2	Gestión de la implementación de la encuesta										
5	ANALISIS DE LOS DATOS										
5.1	Ingreso y verificación de la calidad de los datos										
	Implementación del plan de análisis de datos										
6	USO DE LOS DATOS OBTENIDOS										
6.1	Traducción de los hallazgos a la acción										
6.2	Redacción del informe de la encuesta										
6.3	Difusión de los hallazgos										

Supplementary material 3. Multiple correspondence analysis for additional potential indicator variables explored. (i.e. zone, binary gender, place of work, age)

### Zone

**Figure:**
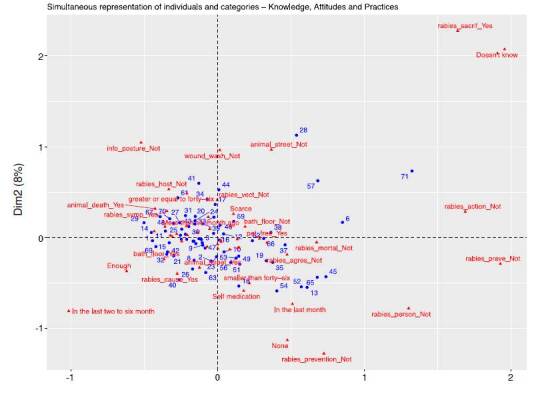

### Binary gender

**Figure:**
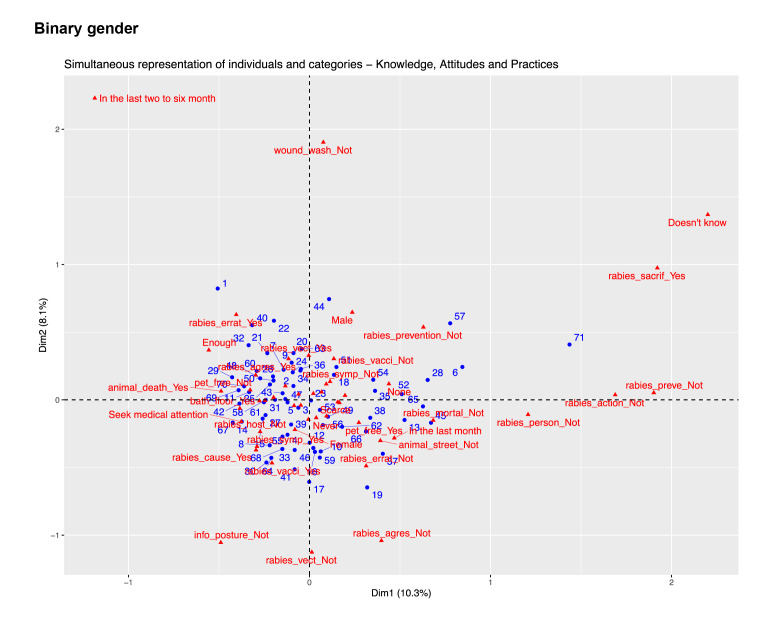

### Place of work

**Figure:**
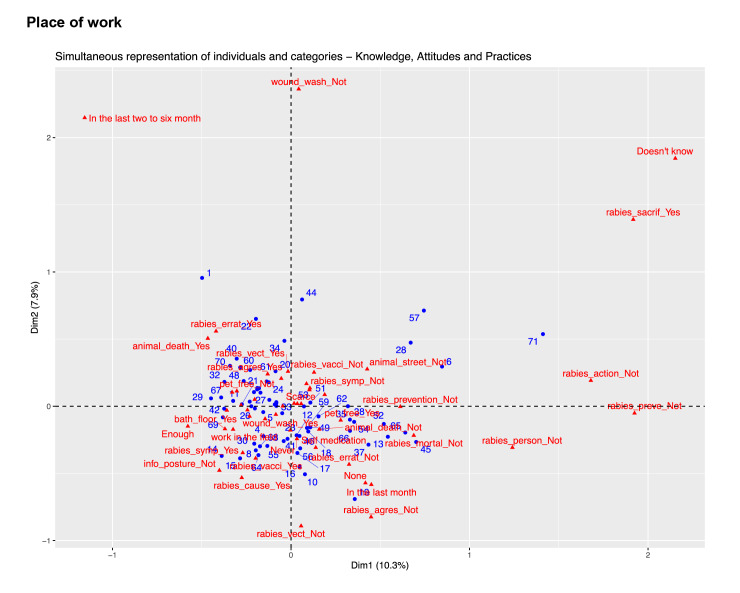

### Age (in years) 

**Figure:**